# Mechanical Performance of Timber Beams Strengthened with Glass Fibre Reinforced Polymer Sheets

**DOI:** 10.3390/ma19163484

**Published:** 2026-08-18

**Authors:** Michał Marcin Bakalarz, Paweł Grzegorz Kossakowski

**Affiliations:** Department of Theory of Structures and Building Information Modeling (BIM), Faculty of Civil Engineering and Architecture, Kielce University of Technology, Al. Tysiaclecia Panstwa Polskiego 7, 25-314 Kielce, Poland; mbakalarz@tu.kielce.pl

**Keywords:** timber beams, GFRP, external strengthening, four-point bending, ductility, stiffness, finite element analysis, transformed cross-section

## Abstract

**Highlights:**

- GFRP sheets were used as passive external reinforcement of pine beams.
- Load-bearing capacity increased by up to 58.20% and ductility by up to 126.14%.
- The effect on bending stiffness was modest, reaching 20% at most.
- Numerical and analytical models were developed and verified against test results.
- Failure shifted from brittle tension to ductile crushing at higher reinforcement ratios.

**Abstract:**

Cost is one of the decisive factors when selecting a fibre type for structural strengthening. This study therefore tested the hypothesis that a low-cost fibre can still provide a substantial improvement in the mechanical performance of strengthened timber beams. Four-point bending tests were carried out on 25 pine beams, comprising an unstrengthened reference series and four series strengthened with glass fibre reinforced polymer (GFRP) sheets, each series consisting of five specimens. The beams had nominal dimensions of 80 mm × 80 mm × 1600 mm. The reinforcement was bonded exclusively to the external surfaces, with a focus on the tension zone. Two variables were examined: the number of sheet layers and the extent of coverage of the timber surface. The reinforcement ratio ranged from 0.38% to 1.15%. The response of the beams was assessed in terms of load-bearing capacity, stiffness, ductility, and failure mode. Bonding three layers of sheet to the bottom face of the beams increased the load-bearing capacity by up to 58.20%. The effect on stiffness was less pronounced, with a maximum increase of 20%, which is attributable to the relatively low elastic modulus of the sheets. However, the ductility of the beams increased significantly (up to 126.14%, based on energy considerations), a result that can be directly attributed to the composite’s high adhesion and high elongation at rupture. The transformed cross-section method and finite element simulations were used to predict the behaviour of the strengthened elements, and both showed good agreement with the test results within the elastic range. It is concluded that GFRP sheets are a rational strengthening solution, although satisfactory effectiveness was obtained only at higher reinforcement ratios; at least two layers are recommended for the configurations tested. The effect of the strengthening configuration on the load-bearing capacity, on the deflection at maximum load and on the ductility was statistically significant, whereas its effect on the bending stiffness was not.

## 1. Introduction

Fibre-reinforced polymer (FRP) composites are used in civil engineering to restore, repair, or reinforce existing and new structures [1,2,3]. Triantafillou and Deskovic [4] were among the precursors in the development of this technique, specifically applying the pretensioned carbon sheets as a reinforcement of wooden members. So far, a wide range of materials and techniques has been developed for this purpose [3]. However, several studies have questioned the economic justification for such solutions, and this observation was the starting point for the present work.

Glass fibre reinforced polymers are, in comparison to carbon fibre reinforced polymers (CFRP) and aramid fibre reinforced polymers (AFRP), a relatively inexpensive material. They are also widely available in a variety of forms and shapes. Their effectiveness is nevertheless open to question, because their mechanical properties are inferior to those of CFRP. Basterra et al. [5] examined duo beams (each composed of two boards with nominal dimensions of 40 mm by 140 mm) with internal GFRP reinforcement placed in a groove in the lower part of the cross-section. Two types of GFRP differing in density were used. Moderate improvements were obtained, of up to 14.7% in bending stiffness and 23% in moment capacity. Morales-Conde et al. [6] investigated the use of GFRP to repair or strengthen timber beams. Tests were carried out on laboratory-scale specimens in two scenarios: repair of decayed beam ends, and repair or strengthening of the midspan region. The technique restored the properties to those of undamaged beams. Gentile et al. [7] studied the behaviour of full-scale sawn Douglas fir beams strengthened with near-surface-mounted (NSM) GFRP bars. Increases in flexural strength of 18% and 46% were achieved for reinforcement ratios of 0.27% and 0.82%, respectively. The proposed technique was subsequently used to strengthen 75 stringers of a bridge near Winnipeg, Manitoba, Canada. NSM bars were also applied by Saribiyik and Akgül [8], who strengthened timber connections and tested them in bending. The bending strength increased by up to 300% relative to adhesively bonded connections. Shahidul Islam et al. [9] used GFRP plates to retrofit timber I-joists with notched flanges and web openings. The load-bearing capacity improved substantially, the magnitude depending on the type of modification, and the technique was sufficient to restore the properties of the control specimens in full. Rostampour-Haftkhani et al. [10] analysed the embedment strength of lag screws in three-ply cross-laminated timber (CLT) made of densified poplar and compared it with CLT reinforced with GFRP in the form of a bidirectional E-glass fabric. Their results showed a significant increase relative to the reference specimens. Schmidt and Fernando [11] demonstrated that GFRP also improves the fire performance of timber structures, identifying two beneficial effects: mechanical protection of the charred timber layers against delamination, and insulation of the deeper layers. Taken together, these studies confirm the potential of GFRP, but they concern mainly internal reinforcement, NSM bars, connections or engineered products; externally bonded GFRP sheets on solid timber beams, and in particular the influence of the number of layers and of the coverage configuration, have received far less attention.

Flexural reinforcement of wooden beams with CFRP materials was discussed by Borri et al. [12]. The maximum increase in flexural capacity achieved in that study was 60% compared to unreinforced, when three layers were applied; while the maximum increase in stiffness was 27.7%. Nowak et al. [13] achieved up to 79% and 32% increases in load-bearing capacity and stiffness for the wooden beams reinforced with internally bonded CFRP plates when compared to the reference. De la Rosa García et al. [14] compared the effectiveness of basalt and carbon fibre fabric as a reinforcement (in “U” type distribution) of solid timber beams. They stated that unidirectional reinforcement with basalt fibre outperformed the application of carbon fibre, implying greater economic effectiveness due to the lower price of basalt fabric when compared to the price of carbon fabric.

The novelty of this study lies in the systematic assessment of low-cost, externally bonded GFRP sheets as reinforcement for solid timber beams, with simultaneous consideration of the number of composite layers and the extent of reinforcement coverage in the tension zone. This approach makes it possible to evaluate not only the general effectiveness of GFRP strengthening, but also the influence of reinforcement configuration on load-bearing capacity, stiffness, ductility and failure mechanisms. While previous studies have confirmed the usefulness of GFRP in timber structures, they have often focused on internal reinforcement, NSM bars, repair of damaged elements, timber connections, I-joists or CLT members. In this context, the present research complements the existing state of knowledge by providing experimental, theoretical and numerical evidence for a simple and economically justified strengthening technique based on externally bonded GFRP sheets. The results therefore extend earlier findings by showing that, despite the lower mechanical properties of glass fibres compared with carbon fibres, appropriately designed GFRP sheet reinforcement can significantly improve the structural performance of timber beams, particularly in terms of load-bearing capacity and ductility.

The aim of the present study was to assess the effectiveness of one of the most economical FRP sheets available commercially when used to strengthen solid pine beams. The variables analysed were the number of reinforcement layers, which determines the cross-sectional reinforcement ratio, and the way in which the reinforcement was distributed over the surface of the beam. The remainder of the paper is organised as follows. Section 2 presents the assumptions of the experimental programme, including the materials, the preparation of the specimens and the test methodology. Section 3 presents the main experimental results; the behaviour of the beams is described in terms of load-bearing capacity, stiffness, ductility and form of failure, and is discussed against the results reported by other authors. Section 4 reports the results of the numerical and analytical analyses. Section 5 summarises the main conclusions and the limitations of the study.

## 2. Materials and Methods

The materials employed in the experimental study were selected on the basis of their availability on the Polish market. Scots pine (*Pinus sylvestris* L.) was chosen because it is widely used in residential construction, for example in roof trusses. Glass fibre reinforced polymer (GFRP) composites are relatively inexpensive and are available in a variety of forms, including bars, sheets and rovings. The reinforcement system was produced by S&P Reinforcement Polska (Malbork, Poland) [15]. The timber was supplied by sawmills operating in the Świętokrzyskie Voivodeship, south-eastern Poland (Kielce, Poland).

### 2.1. Materials

The subjects of the study were pine beams with nominal dimensions of 80 mm by 80 mm by 1600 mm. The beam dimensions were selected on the basis of economic considerations and the capacity of the test rig. The mean bending strength of the timber was 56.22 MPa (standard deviation 7.15 MPa) and the mean global modulus of elasticity in bending was 9.48 GPa (standard deviation 0.82 GPa). Both parameters were determined in accordance with EN 408 [16] on five specimens (described later as BN series). On this basis, the timber corresponded to strength class C20 according to EN 338 [17]. Because the values reported above are mean values obtained from five specimens, they were converted to characteristic values in accordance with EN 384 [18] before the class was assigned. The modulus of elasticity, rather than the bending strength, governed the classification.

Before testing, the timber had been stored for several years in rooms sheltered from the weather, simulating service conditions inside a building. Following the end of the experimental trials, the moisture content of the wood was measured at three points along each beam using a Tanel WRD-100 electrical resistance moisture metre (Tanel Electronics & IT General Partnership, Gliwice, Poland) [19]. The mean moisture content was 9.74% (standard deviation 0.83%), while this value ranged from 8.91% to 10.45% for the BG3 and BGU1, respectively. Before strengthening, the mean density of the timber was 533.98 kg/m^3^, with a standard deviation of 47.04 kg/m^3^. Prior to the reinforcement application, all timber beams were visually inspected and sorted. Specimens with significant natural defects, such as large knots or severe cross-grain, particularly in the assumed tension zone, were rejected to minimise the influence of extreme material flaws on the global structural response.

The selected mechanical and physical properties of the wood are presented in Table 1. Some of these properties were evaluated experimentally, while the rest were estimated using the expressions given in EN 384 [18].

**Figure 1:**
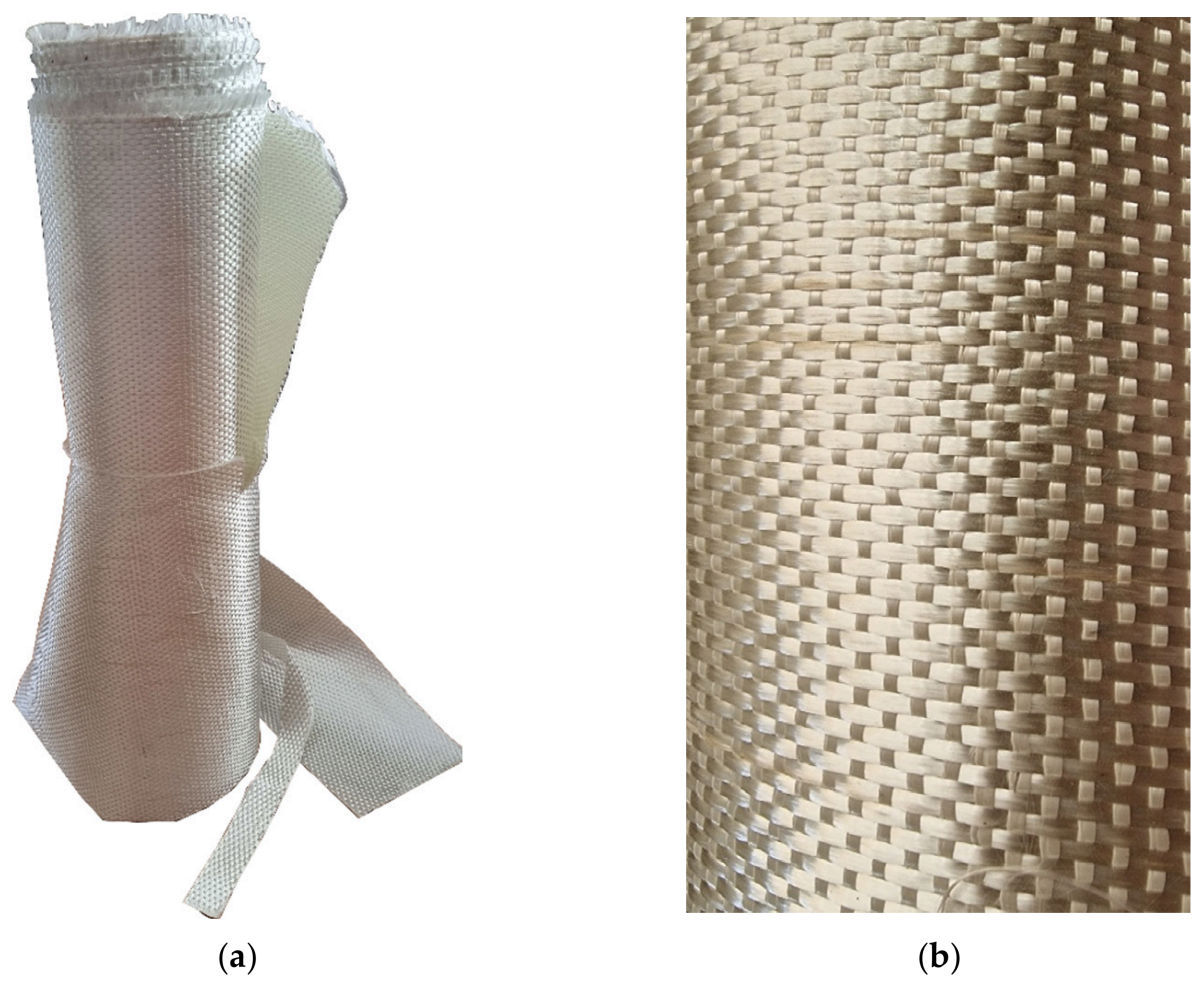
Strengthening system components: (**a**) roll of sheet; (**b**) close-up on sheet.

The beams were strengthened with bi-directional glass fibre reinforced polymer (GFRP) sheets, which were manufactured under the trade name S&P G-Sheet. The glass fibres were arranged in a 90/10 ratio in the main/transverse direction (which accounts for the difference between the mass in the longitudinal direction and the mass per unit area given in Table 2). The longitudinal direction is aligned horizontally while the transverse is aligned vertically in Figure 1b. The sheet was supplied in the form of a roll measuring 670 mm in width. The material was cut to size with scissors. The sheets bonded to the bottom face were 80 mm wide and 1400 mm long. In the U-shaped configuration, the sheets were approximately 160 mm wide and 1400 mm long. The reinforcement configurations investigated are described in Section 2.2. The design thickness of a single layer was 0.308 mm, calculated as the ratio of the areal weight of the fabric to the density of the fibre. The properties of the composite material that have been selected for this study are presented in Table 2, as provided by the manufacturer.

The sheets were bonded using a two-component epoxy resin-based adhesive, S&P Resin 55 HP. The adhesive was supplied in packages of 6 kg. The components were mixed in the recommended proportions for approximately five minutes. The adhesive was then applied to both the sheet and the timber surfaces, and the excess was removed with rubber spatulas. The adhesive consumption was approximately 1 kg per square metre of sheet. The curing time depends on the ambient temperature. The mechanical and physical properties of the adhesive that have been selected are presented in Table 3.

The components of the strengthening system are shown in Figure 1. Before application, the sheets were white and compact. After impregnation with the adhesive, they became partially transparent.

### 2.2. Methods

The unreinforced and reinforced pine beams were subjected to a four-point bending test in accordance with EN 408 [16]. The tests were carried out in the Materials Strength Laboratory of the Kielce University of Technology. The objective of the research was to investigate the impact of the number of layers (increasing number from 1 to 3 layers) and the extent of coverage of the external wood surface (only to the bottom face versus U-type) by the GFRP sheets on the bending behaviour of the beams.

**Figure 2:**
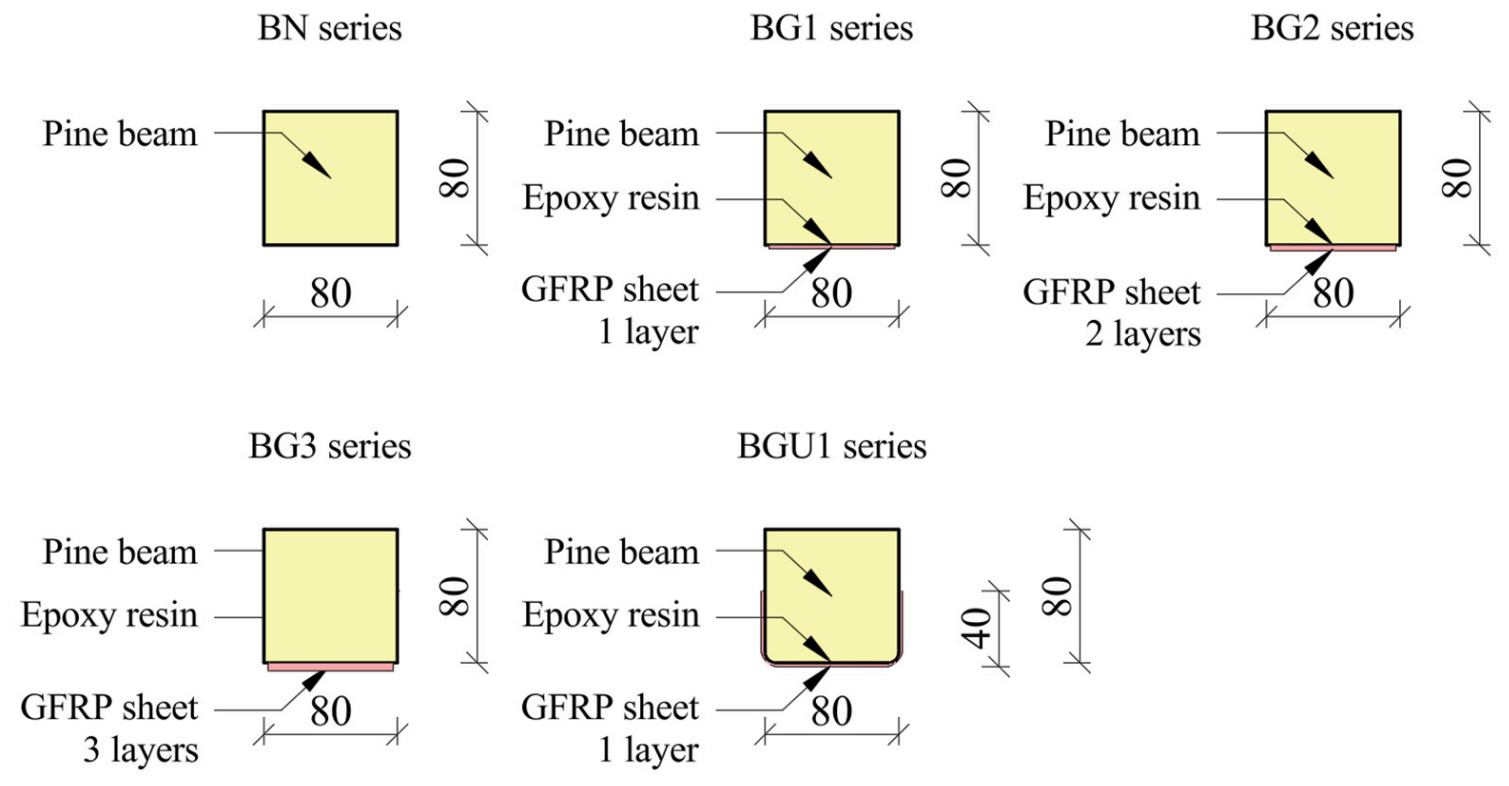
Reinforcement configurations.

In order to achieve the aforementioned objective, five test series were prepared and tested over the full range of behaviour up to failure. The following notations are used to denote the various forms of the object: BN (unreinforced); BG1 (reinforced with one layer of sheet glued to the bottom face); BG2 (reinforced with two layers of sheet glued to the bottom face); BG3 (reinforced with three layers of sheet glued to the bottom face); and BGU1 (reinforced with a U-shaped sheet). Each series comprised five elements. The width of composite sheet applied to the bottom face was 80 mm, while in the U-shape, the total width was 160 mm (40 mm overlap on both side faces). The suffix in each designation denotes the number of the specimen within a test series; for instance, BN1 is unreinforced beam number 1. The reinforcement configurations are shown in Figure 2. The following colour coding was adopted: yellow indicates the timber section and red indicates the composite reinforcement. In each of the aforementioned schemes, the reinforcement was implemented exclusively in the lower half of the unreinforced cross-section’s height. The cross-sectional reinforcement ratio was deliberately kept low, ranging from 0.38% to 1.15%. The thickness of the reinforcement was found to be 0.308 mm, 0.616 mm, and 0.924 mm for one, two, and three layers of reinforcement, respectively. Quartz sand was spread between adjacent composite layers to improve adhesion.

The BN series served as the reference for the present study and comprised the same five unstrengthened beams that were reported by Bakalarz and Kossakowski [22], where they served as the reference for beams strengthened with ultra-high-modulus CFRP sheets. All twenty-five beams considered here and in [22] originated from the same batch of timber and were tested in the same laboratory campaign, under identical conditions and using the same test setup, which allows the results obtained for the different strengthening materials to be compared directly. No results for the strengthened series reported in [22] are reproduced in the present paper, and no figure or table from that paper has been reused.

**Figure 3:**
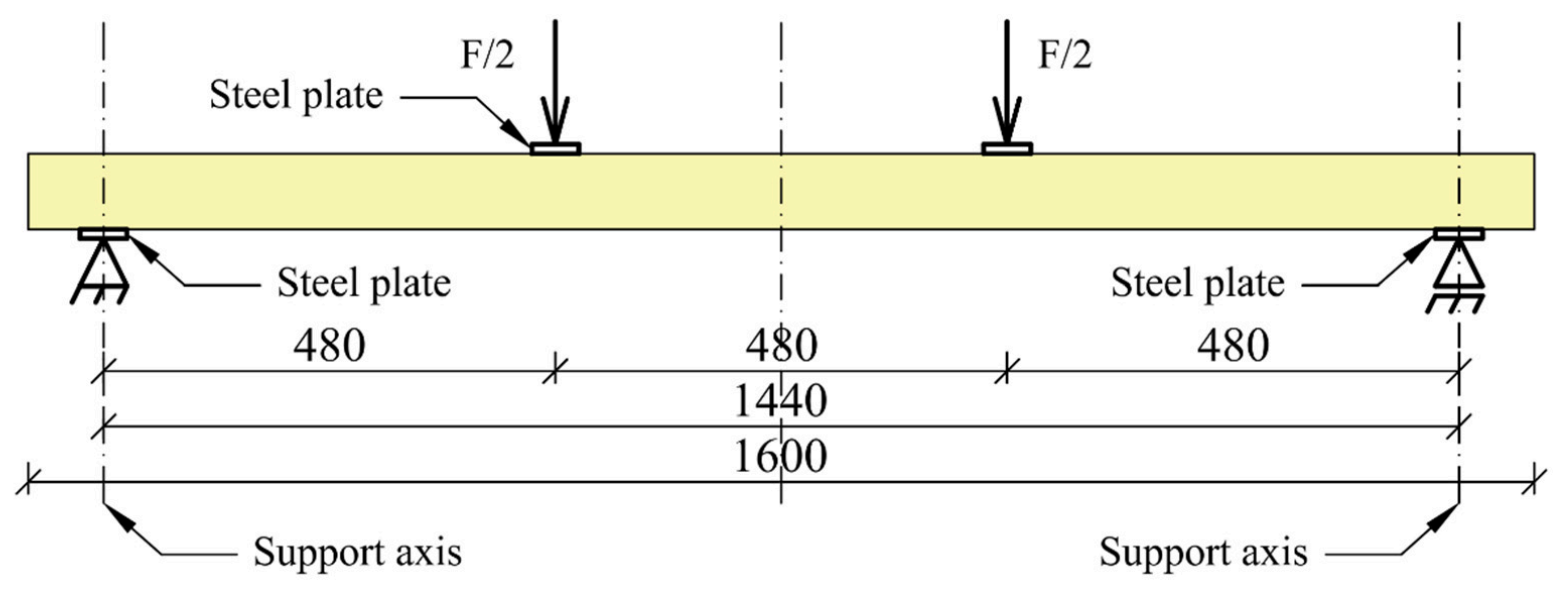
Static scheme configuration.

The adopted static scheme for the so-called four-point bending is illustrated in Figure 3. The span between the support axes was 1440 mm, equal to 18 times the depth of the cross-section. The distance from the support axis to the nearest concentrated force was 480 mm, equal to six times the depth of the cross-section; the two concentrated forces were therefore also 480 mm apart. Steel guide plates 10 mm thick, 40 mm wide, and 200 mm long were used at the supports and at the load application points in order to prevent local indentation of the timber.

**Figure 4:**
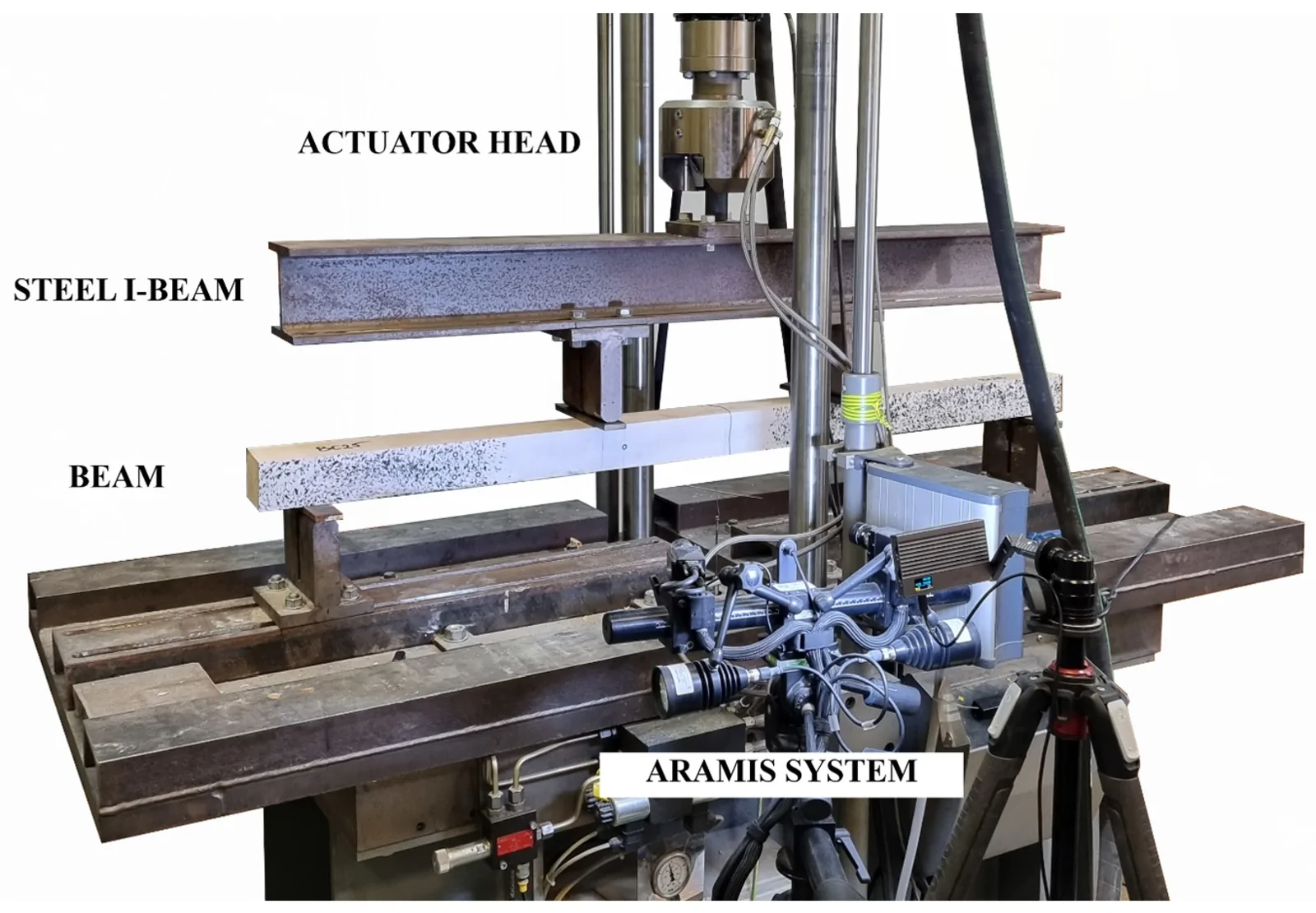
View of the test stand.

The test bench is illustrated in Figure 4. The bending test was conducted on an MTS-322 testing machine, manufactured by MTS Systems Corporation (Eden Prairie, MN, USA), with a load cell capacity of 100 kN. The force generated by the actuator was transmitted to the beam via a steel crosshead constructed from an I-beam. The midspan deflection was measured on the bottom face of the beams with a linear displacement transducer (HBM, Darmstadt, Germany) having a measuring range of 200 mm. Loading was controlled by the displacement rate of the hydraulic actuator. A rate of 7 mm/min was selected so that failure of the unreinforced beams occurred within the time recommended by EN 408 [16]. The tests were conducted until a substantial decrease in force was observed, for instance due to the onset of wood cracking, or until the maximum feasible displacement of the beam on the test stand was attained.

The main response quantities (force and deflection) were recorded at a sampling frequency of 5 Hz. To shorten the processing time, without affecting the results, the curves were subsequently plotted from data down-sampled to 1 Hz.

The bending stiffness of the beams was assessed using the so-called *k* coefficient. The *k* coefficient was evaluated according to the following formula:

*k* = *F*/*u*, (1)

where *F*—loading force in kilonewtons, and *u*—midspan deflection in millimetres.

In order to assess the influence of reinforcement on the bending ductility of beams, a unitless parameter based on the energy absorption measure *µ_E_* was utilised. The *µ_E_* index was evaluated according to the following formula [23]:

*µ_E_* = 1/2 × (*W_tot_*/*W_el_* + 1), (2)

where *W_tot_*—total energy; *W_el_*—elastic energy. The energies were determined as the areas under the load–deflection curves, with characteristic points used as the limits of integration. The point at which the *F*–*u* relationship deviates from linearity was identified as the proportional limit. The end point of integration is marked in the load–deflection curves and differed between specimens. The energies are expressed in joules.

The proportional limit was determined individually for every load–deflection curve. A straight line was fitted by least squares to the portion of the curve lying between 0.1 and 0.4 of the maximum load, that is, over the interval recommended by EN 408 [16] for the determination of the modulus of elasticity in bending. Starting from the upper end of that interval, the recorded load was then compared with the load predicted by the fitted line at the same deflection, and the proportional limit was taken as the first point at which the recorded load fell below the predicted value by more than 5%. This tolerance was adopted so that the criterion would not be triggered by the noise of the measurement or by the local irregularities of the curve caused by the seating of the specimens.

### 2.3. Numerical Model

**Figure 5:**
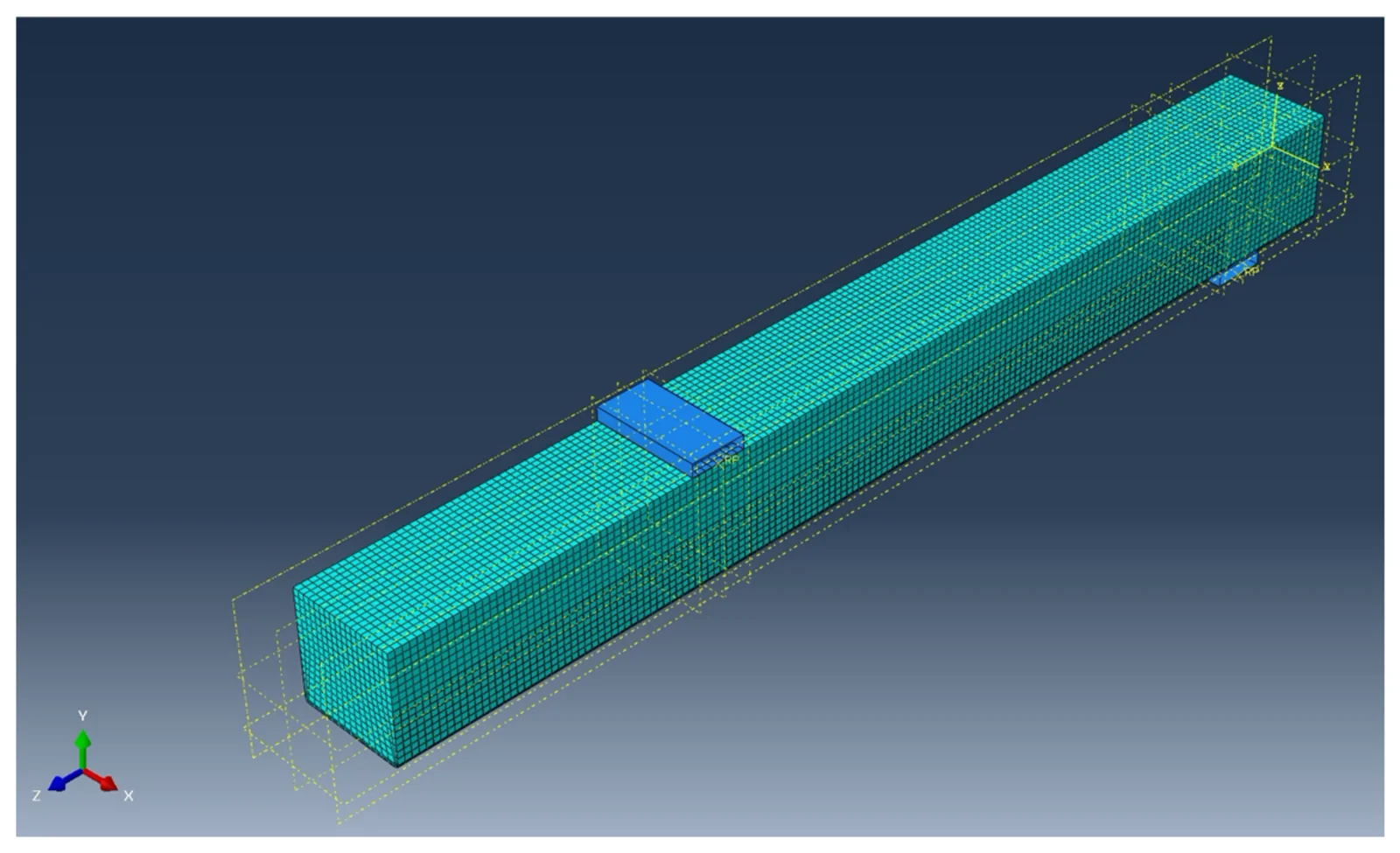
View of meshed BGU1 numerical model.

Bending tests were compared with numerical models created in Abaqus 2017 software (Dassault Systèmes Simulia Corp., Johnston, RI, USA) (Figure 5). The beam part was created as a three-dimensional, deformable body using the profile extrusion method (only half of the model was used due to symmetry). The reinforcement was modelled as a deformable shell part, the number of layers being represented by a corresponding change in shell thickness. Tie constraints were used instead of an adhesive layer under the assumption of a perfect bond—a model limitation justified by the fact that failure was initiated within the wood. A ZSYMM boundary condition was applied to the plane of symmetry. The contact between the steel guide plates and the beam was defined using a surface-to-surface interaction (“hard” contact for the normal behaviour and a friction coefficient of 0.3 for the tangential behaviour). The loading process was conducted by prescribing the displacement of the steel guide plate. S4R and C3D8R element types were used for the reinforcement and the beam, respectively. The approximate global mesh size was 5 mm. The analysis was performed statically (using a Static, General step).

A linear elastic model was assumed for the GFRP sheet. The wood was modelled with the anisotropic yield criterion proposed by Hill [24]. In Abaqus, orthotropic models are defined within the elastic properties using the ‘Engineering constants’ sub-option. The nine independent elastic constants required by this option were derived from the global modulus of elasticity given in Table 1 by means of the ratios between the longitudinal, radial, and tangential directions reported for softwoods by Bodig and Goodman [25] and using the EN 384 [18]—see Table 4.

Comparable modelling strategies for FRP-strengthened timber beams have been reported by Kim and Harris [27], Szczecina [28], Khelifa et al. [29], and Valipour and Crews [30]. The Hill function was determined according to Equation (3) and included in the ‘Potential’ option:

(3) $$f\left(\sigma \right)=\sqrt{F{\left(\sigma_{22}-\sigma_{33}\right)}^{2}+G{\left(\sigma_{33}-\sigma_{11}\right)}^{2}+H{\left(\sigma_{11}-\sigma_{22}\right)}^{2}+2N\tau_{12}^{2}+2M\tau_{13}^{2}+2L\tau_{23}^{2}}$$

where *σ_ij_* and *τ_ij_* correspond to the stress tensors *σ* and the six anisotropy constants were determined using Equations (4) and (5):

(4) $$F=\frac{1}{2}\left(\frac{1}{R_{22}^{2}}+\frac{1}{R_{33}^{2}}-\frac{1}{R_{11}^{2}}\right),\;G=\frac{1}{2}\left(\frac{1}{R_{33}^{2}}+\frac{1}{R_{11}^{2}}-\frac{1}{R_{22}^{2}}\right),H=\frac{1}{2}\left(\frac{1}{R_{11}^{2}}+\frac{1}{R_{22}^{2}}-\frac{1}{R_{33}^{2}}\right),$$

(5) $$N=\frac{3}{{2R}_{12}^{2}},M=\frac{3}{{2R}_{13}^{2}},L=\frac{3}{{2R}_{23}^{2}}.$$

These constants were estimated based on the formulas from the Abaqus manual [26] (6):

(6) $$R_{11}=\frac{{\bar{\sigma }}_{11}}{\sigma^{0}},\;R_{22}=R_{33}=\frac{{\bar{\sigma }}_{22}}{\sigma^{0}}=\frac{{\bar{\sigma }}_{33}}{\sigma^{0}},\;R_{12}=R_{13}=\frac{{\bar{\sigma }}_{12}}{\tau^{0}}=\frac{{\bar{\sigma }}_{13}}{\tau^{0}},\;R_{23}=\frac{{\bar{\sigma }}_{23}}{\tau^{0}},$$

where *τ*^0^ = *σ*^0^/√3. The estimated constants are presented in Table 5 below.

Yield stress was assumed as the compressive strength parallel to the grain. The geometry and material properties were adopted as described in Section 2.1 and Section 2.2.

### 2.4. Transformed Section Method

The stiffness of the composite timber–FRP section was predicted using the transformed cross-section method, in which the FRP reinforcement is replaced by an equivalent area of timber. The properties used are given in Section 2.1. The fundamentals of the method are described in detail elsewhere [31,32,33]; therefore, only a brief outline is given here. The proportional increase in dimensions was determined using the *η* coefficient, which is the ratio of the elastic modulus of the FRP and wood, according to the equation:

(7) $$\eta =\frac{E_{frp}}{E_{m,g,mean}}$$

where *E_frp_*—modulus of elasticity of composite; *E_m_*_,*g*,*mean*_—modulus of elasticity of wood. The basic geometric properties of the transformed section were then evaluated using the fundamental principles of mechanics.

Theoretical value of stiffness coefficient *k_theo_* was evaluated according to the equation:

(8) $$k_{theo}=\frac{2P}{\Delta max(P)}$$

where *P* is half of the total loading force, and Δmax(*P*) is the midspan deflection estimated for the *P* load value. Midspan deflection was estimated according to:

(9) $$\Delta \max\left(P\right)=\frac{Pa}{24E_{m,g,mean}I_{y}}\left(3L^{2}-4a^{2}\right)$$

where *a* is the distance between the support and the nearest load axis, *L* is the support span, and *I_y_* is the second moment of area of the transformed section.

### 2.5. Statistical Analysis

One-way analysis of variance (ANOVA) was used to compare the means of the tested series. The test series, that is, the configuration of the reinforcement, was treated as the independent variable, while the dependent variables were the load-bearing capacity, the bending stiffness coefficient, the ductility index, and the deflection at maximum load. Tukey’s post hoc test was used for pairwise comparisons of the means, and Levene’s test was performed to verify the homogeneity of variance. The normality of the residuals was verified with the Shapiro–Wilk test. The proportion of the total variance explained by the series is reported as *R*^2^ and the effect size as omega squared. Because the number of specimens per series was small, a post hoc power analysis was carried out for every dependent variable: the smallest difference between two means detectable with a power of 80% was computed from the pooled standard deviation, and the number of specimens that would have been required to detect the differences actually observed was estimated. All computations were performed on non-rounded values. The significance level was set at *α* = 0.05. The statistical analysis was conducted using OriginPro 2026 (OriginLab Corporation, Northampton, MA, USA).

## 3. Experimental Results and Discussion

The behaviour of the reinforced beams was evaluated in comparison with that of the reference beams, with particular attention paid to load-bearing capacity, stiffness, ductility and failure mode. The applicability of the transformed cross-section method and of the numerical model for predicting the bending behaviour of the beams was also assessed.

### 3.1. Bending Behaviour

**Figure 6:**
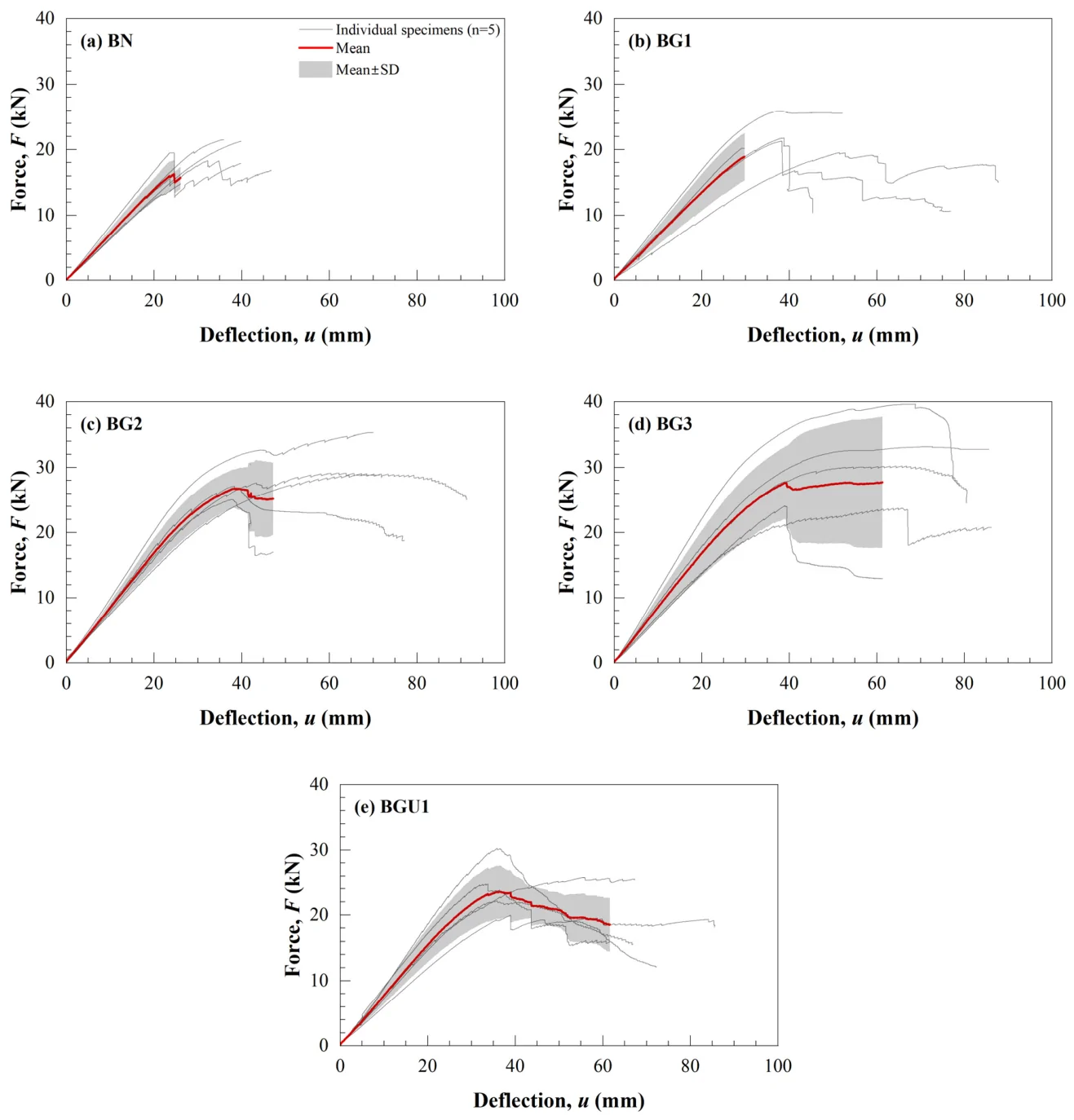
Load versus deflection curves, for series: (**a**) BN; (**b**) BG1; (**c**) BG2; (**d**) BG3; (**e**) BGU1.

The load–deflection paths of the individual beams are plotted as grey lines in Figure 6; each series comprised five beams. The curves are plotted up to the point of failure. Considerable scatter was observed between the individual elements within each series, which is attributable to the inherent heterogeneity and anisotropy of timber as a structural material. For this reason, the mean curve of each series is also shown in every diagram. The mean curves are plotted as red lines, and the range of the mean ± one standard deviation is indicated by the shaded grey bands. The mean curves were obtained by linear interpolation over the common range of deflection; each mean curve therefore terminates at the deflection at which the first beam of the series failed. This point does not correspond to the mean maximum load or to the mean deflection at maximum load, which are reported in Section 3.2. The stepped shape of some curves is attributed to seating of the specimens in the test rig during loading.

The unreinforced beams (Figure 6a) behaved almost linearly from the beginning of the test until failure, and the initial slopes of the curves were similar for all specimens. Sudden drops in load accompanied the formation of cracks. The mean failure load was 19.07 kN, reached at a midspan deflection of 31.73 mm, that is, 272 s after the start of the test. This time lies within the range of 300 ± 120 s required by EN 408 [16].

For the beams reinforced on the bottom face, the range of deformation sustained before failure increased with the number of reinforcement layers. In the initial phase, the slopes of the curves varied more than for the unreinforced beams. A plastic branch developed in the final stage of loading, associated with crushing of the timber in the compression zone (see, Section 3.5). The extent of this plastic branch increased with the reinforcement ratio, which is attributable to the high elongation at rupture of the composite. The mean maximum loads were 21.74 kN, 29.09 kN, and 30.17 kN for series BG1, BG2, and BG3, corresponding to increases of 14.0%, 52.5%, and 58.2% relative to the unreinforced beams. The gain obtained by adding the third layer was small (3.7% with respect to BG2), which indicates that the effectiveness of further layers diminishes rapidly.

The U-reinforced beams behaved in a similar manner, but reached a mean maximum load of only 24.57 kN, and their plastic branch was markedly shorter than that of series BG2 and BG3. After the peak load, the force decreased approximately linearly. This comparison is particularly informative because series BGU1 and BG2 have the same reinforcement ratio of 0.77%, and differ only in the distribution of the sheet over the cross-section. Concentrating the reinforcement on the bottom face gave a mean capacity 18.4% higher than distributing the same amount of composite over the bottom face and the lower part of the side faces, although this difference did not reach statistical significance (see Section 3.2).

### 3.2. Peak Load and Deflection at Peak Load Values

The detailed test results are presented in Table 6 and Table 7, which give the maximum load and the corresponding midspan deflection. The individual results are listed together with the mean values, the increases relative to the reference series, and basic measures of dispersion. It should be noted that in some specimens the deflection given in Table 6 does not correspond to the point of failure, because the peak load occurred before final failure.

Both the load-bearing capacity and the deflection at maximum load increased with the number of reinforcement layers, but the relationship was clearly non-linear. Bonding a single layer increased the load-bearing capacity by 14.00% and the deflection by 23.60%. The second layer produced by far the largest gain, raising these values to 52.54% and 76.32%. The third layer, however, added only 5.66 percentage points in load-bearing capacity, although it still increased the deflection at maximum load by a further 18.98 percentage points. That effect was also discussed by de la Rosa García et al. [14]—“The application of two reinforcement layers did not imply an increase in the carrying capacity of the beams”. The effectiveness of successive layers in terms of strength therefore diminishes rapidly, whereas their contribution to deformability persists. This behaviour is attributed to the progressive mobilisation of the strength reserve of the timber in the compression zone, accompanied by the change in failure mode described in Section 3.5. Further increases in capacity would require the compression zone to be strengthened as well, since it governs failure in these configurations. Series BG2 and BGU1 deserve particular attention, because the same quantity of GFRP sheet was used in both and they differ only in its distribution over the cross-section. The U-shaped configuration achieved an increase in load-bearing capacity of 28.84%, that is only about 55% of the increase obtained for series BG2. This is consistent with elementary bending mechanics, since part of the sheet is placed on the side faces, closer to the centroid of the cross-section, where its contribution to the second moment of area is smaller. The deflection at maximum load behaved in the same way, reaching 39.93 mm for series BGU1 compared with 55.95 mm for series BG2. Consequently, where the ultimate load governs the design, concentrating the reinforcement on the bottom face of the beam is the more effective solution. The comparison of the two configurations in terms of stiffness is discussed in Section 3.3.

**Figure 7:**
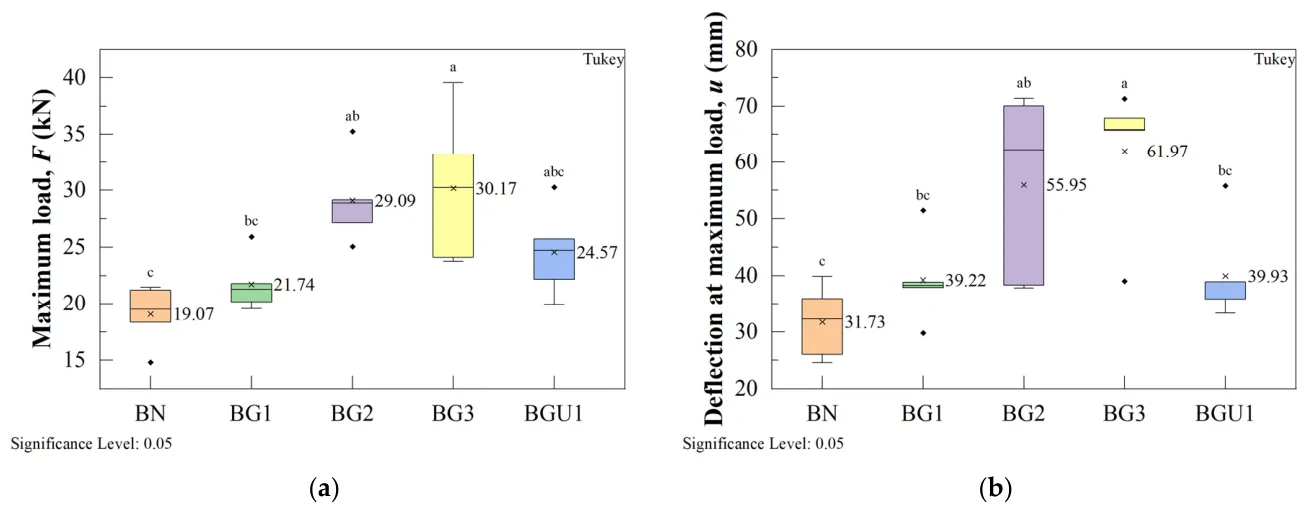
Box charts for means of: (**a**) maximum loading force, and (**b**) corresponding deflection.

A one-way analysis of variance (ANOVA) was carried out to assess the influence of the reinforcement configuration on the maximum load, using five test series (BN, BG1, BG2, BG3 and BGU1) with five specimens each (*n* = 5 per series, *N* = 25). The significance level was set at *α* = 0.05. The assumptions of the analysis were verified beforehand. Levene’s test indicated homogeneous variances, *F*(4, 20) = 1.41, *p* = 0.2663, and the Shapiro–Wilk test performed on the residuals confirmed that they did not depart from normality, *W* = 0.969, *p* = 0.630. The effect of the test series on the maximum load was statistically significant, *F*(4, 20) = 6.40, *p* = 0.0017. The series accounted for 56.15% of the total variance (*R*^2^ = 0.5615), and the effect size was large (*ω*^2^ = 0.4637). Post hoc pairwise comparisons using Tukey’s HSD test showed that series BG2 and BG3 carried significantly higher loads than the unreinforced series BN, the mean differences being 10.03 kN (*p* = 0.0090) and 11.11 kN (*p* = 0.0036), respectively. Series BG3 also differed significantly from BG1, the mean difference being 8.43 kN (*p* = 0.0331). None of the remaining comparisons reached significance. The homogeneous groups obtained from the test are given in Table 6 (see also Figure 7a for the graphical representation). Three results deserve particular attention. First, series BG2 and BG3 were statistically indistinguishable (mean difference 1.08 kN, *p* = 0.9937), which shows that the third layer of sheet produced no demonstrable gain in load-bearing capacity over two layers. Second, the increase obtained with a single layer (series BG1, 14.0%) was not statistically significant (*p* = 0.8480), so the effectiveness of one layer could not be demonstrated for the sample size used. Third, the U-shaped configuration did not differ significantly either from the unreinforced series (*p* = 0.2670) or from series BG2, which has the same reinforcement ratio (*p* = 0.4490); the difference between the two layouts observed in the mean values therefore represents a trend rather than a demonstrated effect. These non-significant results have to be interpreted in the light of the statistical power of the test. With five specimens per series and a pooled standard deviation of 4.18 kN, the smallest difference detectable with a power of 80% is 10.27 kN, that is, 54% of the mean load of the unreinforced beams. The absence of significance should therefore be read as an absence of evidence rather than as evidence of absence, and the limited sample size is acknowledged as a limitation of the study.

The same procedure was applied to the deflection at maximum load. The assumptions were verified beforehand. Levene’s test did not indicate significant differences between the variances, *F*(4, 20) = 2.43, *p* = 0.0812, and the Shapiro–Wilk test performed on the residuals confirmed that they did not depart from normality, *W* = 0.964, *p* = 0.495. The effect of the test series on the deflection at maximum load was statistically significant, *F*(4, 20) = 6.32, *p* = 0.0019. The series accounted for 55.82% of the total variance (*R*^2^ = 0.5582), and the effect size was large (*ω*^2^ = 0.4597), which closely mirrors the values obtained for the maximum load. Tukey’s HSD test showed that series BG2 and BG3 deflected significantly more than the unreinforced series BN, the mean differences being 24.22 mm (*p* = 0.0211) and 30.24 mm (*p* = 0.0032), respectively. Series BG3 also differed significantly from BG1, the mean difference being 22.75 mm (*p* = 0.0327), and from BGU1, the mean difference being 22.04 mm (*p* = 0.0403). The homogeneous groups are given in Table 7 (see also Figure 7b for the graphical representation). Two aspects of these results are of particular interest. First, series BG2 and BG3 could not be distinguished from each other (mean difference 6.02 mm, *p* = 0.9129), which is consistent with the behaviour observed for the maximum load and confirms that the third layer of sheet did not produce a demonstrable improvement in either quantity. Second, series BG1 and BGU1 gave practically identical deflections, 39.22 mm and 39.93 mm (mean difference 0.71 mm, *p* > 0.999), even though the reinforcement ratio of series BGU1 is twice that of series BG1. The U-shaped configuration thus behaved, in terms of deformability, as if only half of the composite were structurally effective, which is consistent with the smaller lever arm of the sheet bonded to the side faces of the beam. As in the case of the maximum load, the non-significant results have to be read in the light of the statistical power of the test. With five specimens per series and a pooled standard deviation of 11.25 mm, the smallest difference detectable with a power of 80% is 27.65 mm, that is, 87% of the mean deflection of the unreinforced beams. The comparison of series BG2 and BGU1, which have the same reinforcement ratio and differ only in the distribution of the sheet, gave a mean difference of 16.02 mm that did not reach significance (*p* = 0.2022). The absence of significance should therefore be interpreted as an absence of evidence rather than as evidence of absence.

Because the reference series is common to both studies, the effectiveness of the glass sheets can be compared directly with that of the ultra-high-modulus carbon sheets reported in [22]. The GFRP sheets increased the load-bearing capacity by up to 58.20%, whereas the UHM CFRP sheets gave a maximum increase of 9%; conversely, the CFRP sheets produced a larger gain in bending stiffness, 34% against 20%. This contrast follows from the difference in the modulus of elasticity and the elongation at rupture of the two materials.

### 3.3. Bending Stiffness (Experiment)

The mean values of the bending stiffness coefficient *k* and the associated statistical data are given in Table 8. The coefficient was determined over the load interval from 0.1 to 0.4 of the maximum force, which corresponds to the range of stable linear response and is also the interval recommended by EN 408 [16] for the determination of the modulus of elasticity in bending. The mean values were 0.71, 0.68, 0.85, 0.85 and 0.76 kN/mm for series BN, BG1, BG2, BG3 and BGU1, respectively, with coefficients of variation between 10.3% and 20.5%, that is, a dispersion comparable to that obtained for the load-bearing capacity. The mean values do not follow the reinforcement ratio monotonically. Series BG1, with a single layer of sheet, gave a mean stiffness 3.14% lower than that of the unreinforced beams, whereas series BG2 and BG3 gave increases of 19.64% and 19.87%, that is, practically the same value despite the third layer of sheet. The U-shaped configuration reached an increase of 8.21%, which is less than half of the increase obtained for series BG2 even though the reinforcement ratio of the two series is identical. The largest observed increase in bending stiffness was therefore of the order of 20%, and it was reached with two layers of sheet. The modest magnitude of these effects reflects the low modulus of elasticity of the glass fibre sheet relative to that of carbon sheet [22], so that the composite contributes far less to the stiffness of the section than to its load-bearing capacity; the natural variability of the timber further obscures the effect.

The bending stiffness of series BG1, which was 3.14% lower than that of the reference series, requires additional comment, since the addition of reinforcement cannot in itself reduce the stiffness of a cross-section. The difference is smaller than the scatter within the series and is not statistically significant, all five series having been assigned to a single homogeneous group. The mean of series BG1 is drawn down by the last specimen of that series, for which *k* = 0.48 kN/mm. Formal outlier tests do not identify this value as an outlier at the 5% level, and no procedural reason for its exclusion was found, so it has been retained in the analysis. The values recorded for the remaining four specimens ranged from 0.66 to 0.87 kN/mm and bracket the mean of series BN; omitting the lowest specimen would give a mean of 0.738 kN/mm, that is an increase of 4.2% over the reference series. An increase in this order is what the modulus of elasticity of the glass sheet, low in comparison with that of carbon reinforcement, permits at a reinforcement ratio of 0.38%, and an effect of this size cannot be resolved against a coefficient of variation of 20.5% within the series. The apparent reduction is therefore attributed to the natural variability of the timber rather than to any physical effect.

The magnitude of the gain in bending stiffness obtained here is consistent with the values reported in the literature for comparable reinforcement ratios. Fiorelli and Dias [34] recorded increases of between 15% and 30% for beams reinforced with 1.0% of glass fibre and 0.4% of carbon fibre respectively, and the increase of 19.87% obtained here for series BG3 at a reinforcement ratio of 1.15% falls within that range. Raftery and Harte [35] likewise reported only a moderate improvement in both the global and the local modulus of elasticity of glued laminated beams reinforced with glass FRP plates. The gain is governed primarily by the modulus of elasticity of the fibre rather than by the amount of composite. Andor et al. [36] obtained an increase of approximately 30% in load-bearing capacity but of only about 16% in elastic stiffness for Norway spruce beams reinforced with carbon fabric, the same disproportion between the two quantities as that reported by Nowak et al. [13]. The authors of [36] also found that one and two layers of sheet bonded to the bottom face gave similar increases in stiffness, that is, that the second layer added little. The present results show the same saturation, although it occurs one layer later: series BG2 and BG3 gave practically identical increases of 19.64% and 19.87%, whereas the step from one to two layers was still substantial here. Saad and Lengyel [37] examined this discrepancy specifically and concluded that the stiffness enhancement measured in tests is systematically smaller than the analytical prediction, the difference arising from imperfect composite action at the timber–composite interface and from the difference between the moduli of elasticity of timber in tension and in compression, neither of which is accounted for in the transformed cross-section approach.

**Figure 8:**
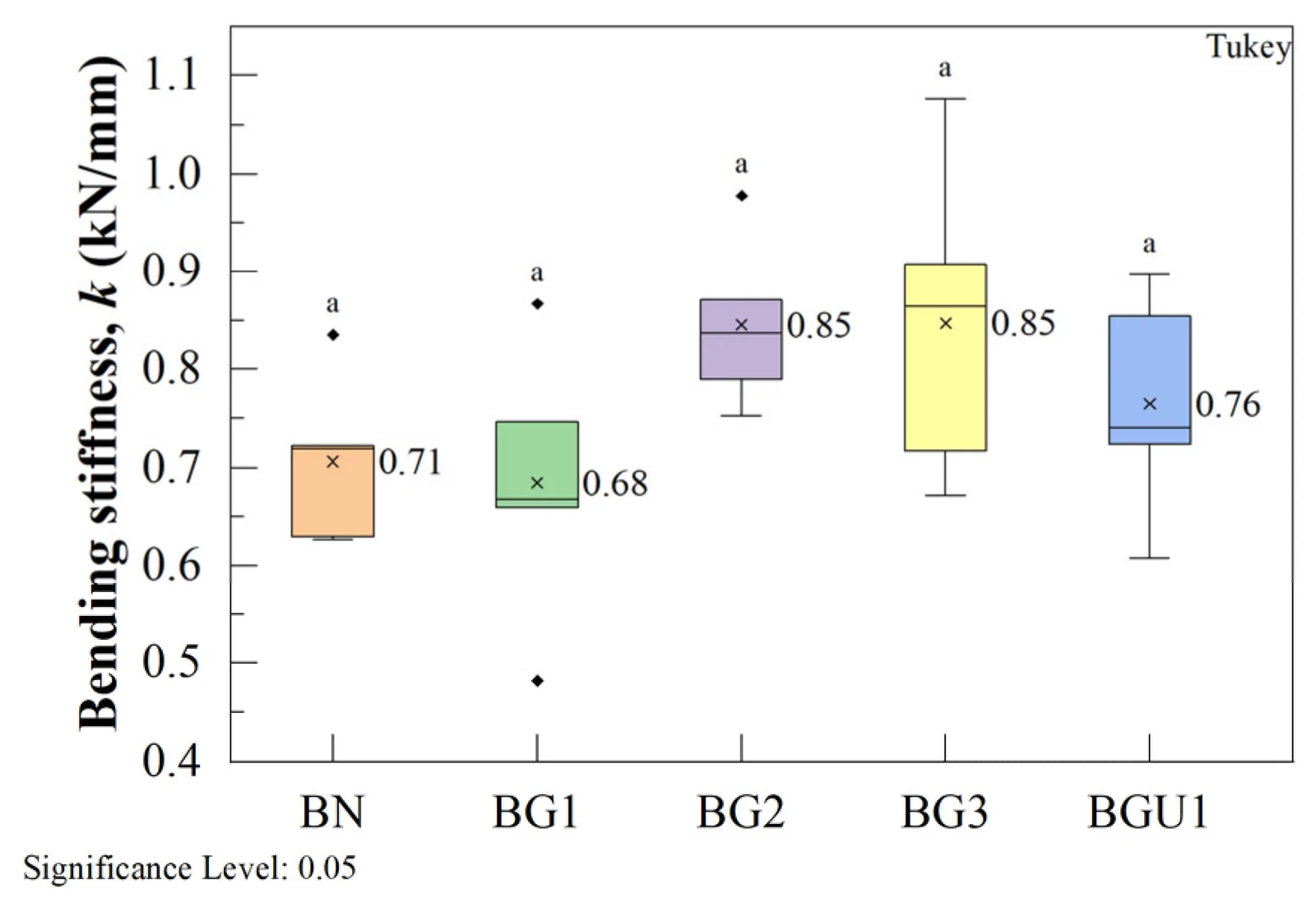
Box charts for means of stiffness coefficient *k*.

The assumptions of the analysis of variance were verified beforehand. Levene’s test did not indicate significant differences between the variances, *F*(4, 20) = 0.68, *p* = 0.6140, and the Shapiro–Wilk test performed on the residuals confirmed that they did not depart from normality, *W* = 0.985, *p* = 0.961. In contrast to the maximum load and the deflection at maximum load, the effect of the test series on the bending stiffness coefficient was not statistically significant, *F*(4, 20) = 1.95, *p* = 0.1415. The series accounted for 28.05% of the total variance (*R*^2^ = 0.2805), and the effect size was moderate (*ω*^2^ = 0.1318). Tukey’s HSD test accordingly revealed no significant pairwise differences and assigned all five series to a single homogeneous group (Figure 8). This outcome has to be interpreted in the light of the statistical power of the test. With five specimens per series and a pooled standard deviation of 0.121 kN/mm, the smallest difference detectable with a power of 80% is 0.30 kN/mm, that is, 42% of the mean stiffness of the unreinforced beams, whereas the largest difference actually observed was 0.163 kN/mm. The moderate effect size indicates that a real but small effect is likely to be present; the experiment simply did not have sufficient power to demonstrate it. The absence of significance should therefore be interpreted as an absence of evidence rather than as evidence of absence, and the increases in stiffness reported above should be regarded as trends that require confirmation on a larger sample.

Table 9 gives the loads corresponding to the deflection limits recommended in EN 1995-1-1 [38], *L*/300 and *L*/250, which for the span of 1440 mm used here correspond to midspan deflections of 4.80 mm and 5.76 mm, respectively. The values were read directly from the *F*–*u* curves at these deflections and may therefore differ slightly from those obtained by multiplying the bending stiffness coefficient *k* by the limiting deflection. The two sets of results are nevertheless consistent: for every series the increase relative to the reference beams is practically the same at both limits, which confirms that the response of the beams is still linear at this level of loading.

Series BG1 carried a slightly lower load than the reference series at both limits, by 1.18% and 1.82%, respectively. This follows directly from the reduced mean stiffness of that series and has the same explanation, namely the variability of the timber rather than any effect of the reinforcement. Series BG2 and BG3 behaved almost identically, both gaining approximately 20%, whereas series BGU1 gained about 7%. The ranking of the configurations under the serviceability criterion is therefore the same as their ranking by bending stiffness, and in particular series BG2 sustains a service load approximately 13% higher than series BGU1 at the same reinforcement ratio of 0.77%. It should be borne in mind, however, that the differences in stiffness on which these loads depend were not statistically significant (*p* = 0.1415), so that the comparison indicates a trend rather than a demonstrated effect.

### 3.4. Energy-Based Bending Ductility

The energy-based ductility index increases when the total energy absorbed by the beam rises while the elastic energy remains unchanged or decreases; a higher maximum load combined with a larger deformation before failure therefore produces a higher index. The results are summarised in Table 10. The ductility index increased with the reinforcement ratio, although the increase was not linear. The largest mean increase, 126.14%, was obtained for series BG3, which had the highest reinforcement ratio. Series BG2 and BGU1, which have an identical reinforcement ratio of 0.77% and differ only in the distribution of the sheet over the cross-section, gave increases of 69.90% and 84.63%; the difference between them was the smallest recorded for any pair of series in this analysis (*p* = 0.9896), so the way in which the side faces are covered had no demonstrable effect on the ductility. The smallest increase, 35.86%, was obtained for series BG1. The coefficients of variation ranged from 22.1% for series BG2 to 37.7% for series BG1 and exceeded those obtained for all the other parameters analysed. The ductility index was thus the most variable of the quantities examined, which follows from its definition as a ratio of two energies, each of which carries its own experimental scatter, and from the additional uncertainty introduced by the determination of the proportional limit.

The value of 1.00 for the fourth beam from the BG1 series corresponds to the situation in which the elastic and total energy values were equal. In such a situation, the beam failed before the occurrence of the plastic branch—the adopted methodology was not able to capture the proportionality limit value before the peak load. This beam failed by shear along the grain (as stated in Table 11).

The proportional limit was determined here by the departure of the curve from the initial straight line, whereas a fixed threshold of 0.7 *F_max_* was adopted in [22]. The ductility indices reported for the BN series therefore differ between the two papers although they were computed from the same experimental curves. The present procedure gives a lower proportional limit and consequently a smaller elastic energy, so that the indices reported here are not directly comparable with those obtained using a fixed threshold.

**Figure 9:**
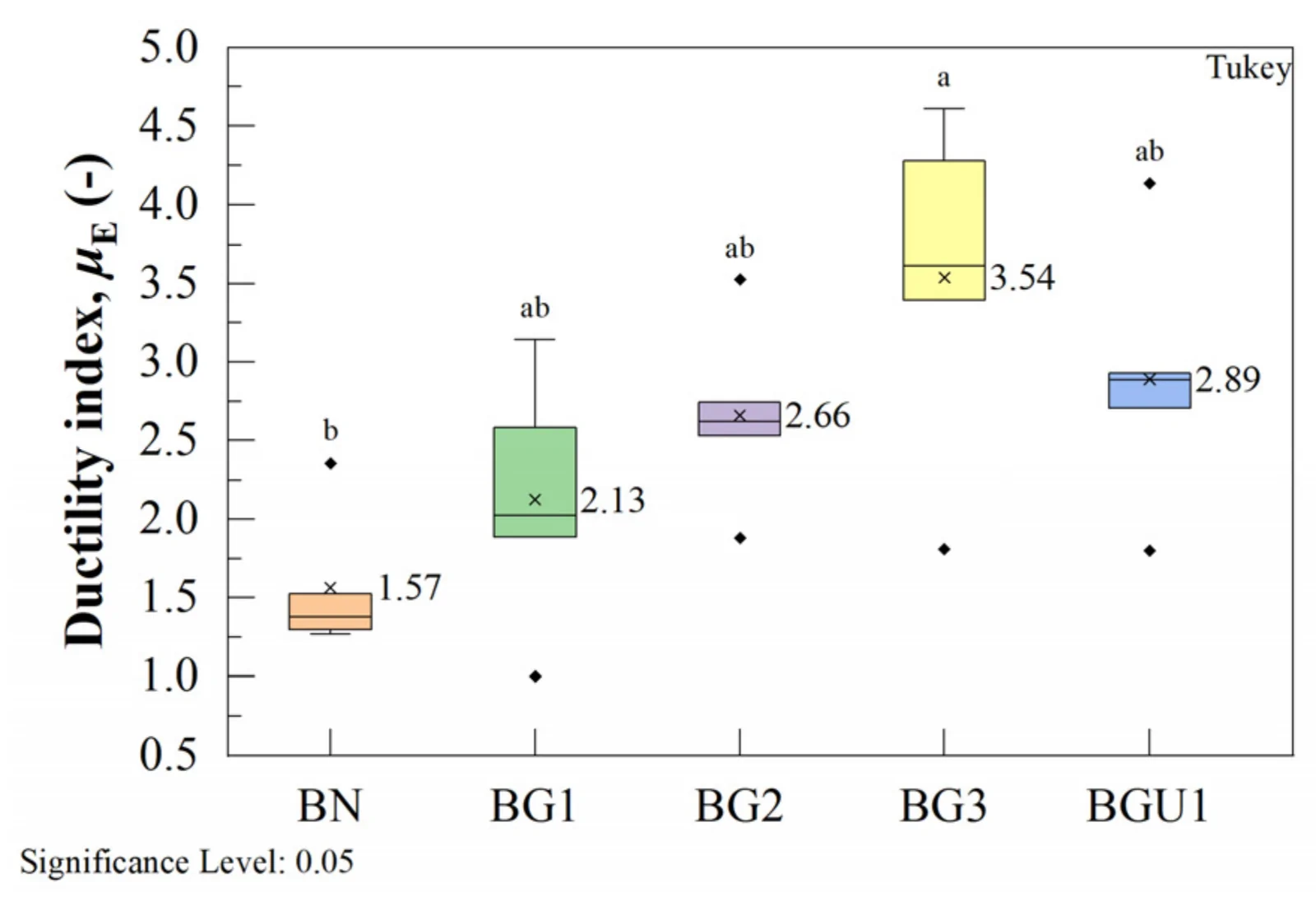
Box charts for means of ductility index with Tukey’s groups.

The assumptions of the analysis of variance were verified beforehand. Levene’s test did not indicate significant differences between the variances, *F*(4, 20) = 0.57, *p* = 0.6882, and the Shapiro–Wilk test performed on the residuals confirmed that they did not depart from normality, *W* = 0.941, *p* = 0.155. The effect of the test series on the ductility index was statistically significant, *F*(4, 20) = 4.59, *p* = 0.0086. The series accounted for 47.89% of the total variance (*R*^2^ = 0.4789), and the effect size was large (*ω*^2^ = 0.3652). Tukey’s HSD test identified only one significant pairwise difference: series BG3, with three layers of sheet, had a higher mean than the unreinforced series BN (*p* = 0.0058). The difference between series BG3 and BG1 approached significance (*p* = 0.0664), as did that between series BGU1 and BN (*p* = 0.0942), whereas series BG2 did not differ significantly from BN (*p* = 0.2170) and series BG3 did not differ significantly from BG2 (*p* = 0.4133). The homogeneous groups are given in Table 10 and the comparison intervals in Figure 9. The non-significant results have to be read in the light of the statistical power of the test, which is lower here than for any other parameter examined. With five specimens per series and a pooled standard deviation of 0.784, the smallest difference detectable with a power of 80% is 1.93, that is, 123% of the mean index of the unreinforced beams. Only the comparison between series BG3 and BN involved a difference of this magnitude, which explains why it was the only one to reach significance. The remaining comparisons should therefore be regarded as inconclusive rather than as evidence that the corresponding differences do not exist.

### 3.5. Failure Modes

**Figure 10:**
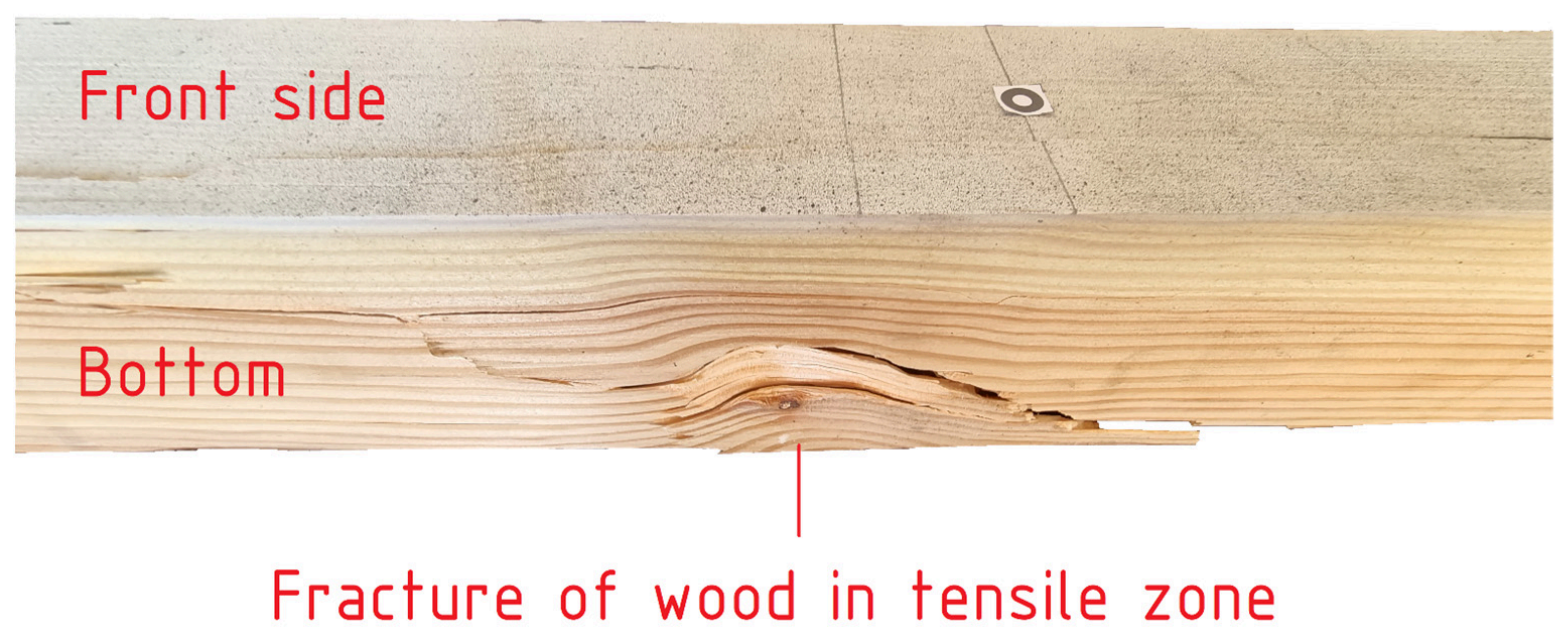
Typical failure mode of unstrengthened beam.

The unreinforced beams failed by cracking of the timber in the tension zone. Failure was sudden and gave no visible warning. A representative example (beam BN1) is shown in Figure 10; all five beams of series BN failed in this manner.

**Figure 11:**
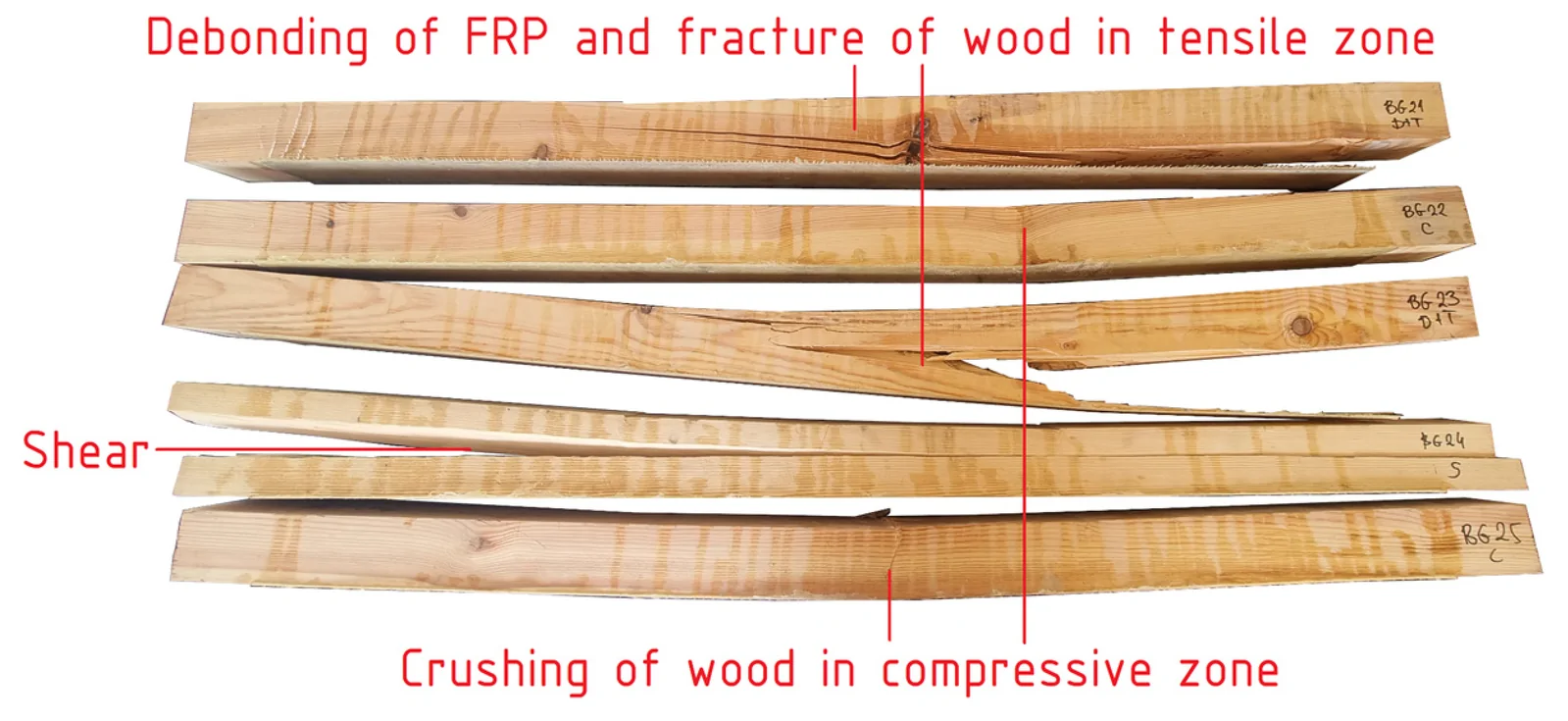
Failure modes observed in the strengthened beams, illustrated on series BG2 (specimens BG21 to BG25, from top to bottom).

The strengthened beams showed a much wider range of behaviour. In every case failure was initiated in the timber by exceeding its tensile, compressive, or shear strength; in no case was rupture of the composite observed. This last point is important, because it indicates that the tensile capacity of the sheet was never fully mobilised, which is consistent with the diminishing effectiveness of successive layers reported in Section 3.2. In some specimens the sheet became detached from the timber surface, and in most of these specimens the detachment followed the propagation of a crack in the tension zone rather than preceding it; in the two beams of series BG1 that failed by crushing, it followed the development of the compression failure. The range of failure modes observed within a single series is illustrated in Figure 11 for series BG2, in which three distinct modes occurred among five nominally identical beams: cracking of the timber in the tension zone accompanied by debonding of the sheet, crushing of the timber in the compression zone, and shear failure along the grain. The number of specimens exhibiting each mode in each series is summarised in Table 11.

The number of specimens in which debonding was recorded fell from five out of five in series BG1 to two out of five in series BG2 and BG3, and no debonding at all was observed in series BGU1. Over the same range, crushing of the timber in the compression zone became progressively more frequent, occurring in all five specimens of series BG3 and BGU1. In the beams with the sheet bonded to the bottom face and to the side faces (series BGU1), failure occurred by crushing of the timber in the compression zone. This suggests that covering the side faces may be beneficial for reasons unrelated to load-bearing capacity: it makes the mode of failure more uniform by confining the natural defects of the timber, which improves the predictability of the response, and it protects the sheet against debonding by distributing the interfacial shear stresses over a larger area. The minimum extent of coverage required to maintain this behaviour was not investigated here and merits further study.

The transition from failure by cracking of the timber in the tension zone to failure by crushing in the compression zone is characteristic of strengthened timber members and has been reported in the literature for a variety of strengthening strategies [12,13,27]. It reflects the fact that the reinforcement relieves the tension zone, so that the compressive capacity of the timber, which is accompanied by a substantial plastic deformation, becomes decisive.

## 4. Numerical and Theoretical Findings

### 4.1. Load–Deflection Paths

**Figure 12:**
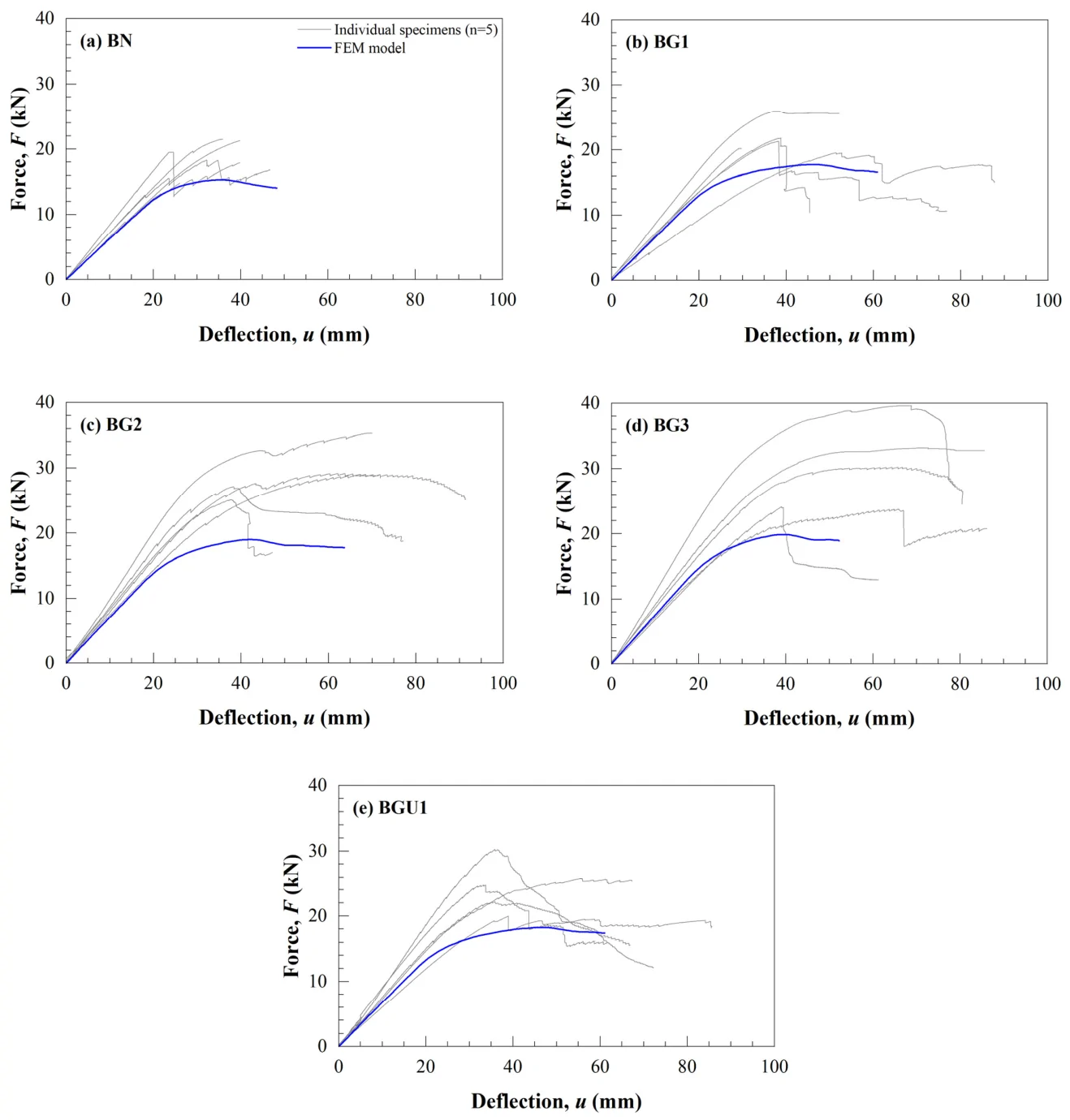
Numerical and theoretical curves plotted against experimental results, for series: (**a**) BN; (**b**) BG1; (**c**) BG2; (**d**) BG3; (**e**) BGU1.

The equilibrium paths obtained from the numerical simulations are plotted against the experimental curves in Figure 12, where they are drawn in blue and labelled FEM. In most of the series, the initial part of the numerical curve lies close to the least stiff beam of the series, and therefore below the mean curve shown in Figure 6. Over the full range of the test, however, the numerical curves reproduce the overall trend of each series well. The peak loads returned by the model were lower than those measured. These discrepancies are attributed to the natural variability of the timber and to the conservative values assumed for the yield stress and for the moduli of elasticity in the radial and tangential directions, all of which were estimated from the relations given in EN 384 [18]. A model based on these parameters therefore yields results that are conservative and, from an engineering point of view, safe.

### 4.2. Bending Stiffness

The values of the bending stiffness coefficient *k* predicted by the transformed cross-section method and by the numerical model are given in Table 12. Every predicted value was lower than the corresponding experimental mean. The relative error ranged from −1.5% to −14.1% for the transformed cross-section method and from −2.9% to −16.5% for the numerical model. The two sets of predictions were close to each other, and no appreciable difference between the two approaches was found.

### 4.3. Peak Loads

The maximum recorded load values from the numerical curves are given in Table 13 next to the experimental mean of the corresponding series. Every model underestimated the peak load, with the relative error ranging from −34.8% to −18.4%. In order to examine the influence of the assumed yield stress on the accuracy of the prediction, a modified model, referred to below as FEM mod, was also analysed. In this model, the compressive strength parallel to the grain was increased by a factor of approximately 4/3. The reason for this modification is that the value adopted initially had been estimated from the relations given in EN 384, which return a strength close to the characteristic level, whereas a model intended to reproduce the mean behaviour of a series requires the mean strength of the batch as input. The factor applied brings the assumed strength to about 41 MPa, a value typical of the mean compressive strength of pine of the density measured here. The modification reduced the error appreciably, the range becoming +0.8% to −16.8%.

## 5. Conclusions

This paper presents the results of an experimental study on the strengthening of solid pine beams with glass fibre reinforced polymer sheets. The influence of the number of layers and of the extent of surface coverage on the load-bearing capacity, stiffness, ductility, and failure mode was examined. The following conclusions were drawn.

- The reinforcement improved the mechanical performance of the beams, but the improvement was clearly non-linear. Single-layer strengthening increased the load-bearing capacity by 14.00% and the deflection at maximum load by 23.60%, neither of which was statistically significant. Two layers raised these values to 52.54% and 76.32%, and three layers to 58.20% and 95.30%. The third layer therefore contributed only 5.66 percentage points of additional capacity, and series BG2 and BG3 could not be distinguished statistically (*p* = 0.9937). Series BG3 differed significantly from BN (*p* = 0.0036) and BG1 (*p* = 0.0331), and series BG2 differed significantly from BN (*p* = 0.0090).
- The largest increase in bending stiffness was approximately 20%, obtained for series BG2 and BG3. The effect of the reinforcement on this parameter was not statistically significant (*p* = 0.1415), although the moderate effect size (*ω*^2^ = 0.1318) suggests that a real but small effect is present, which the sample size was insufficient to demonstrate. The limited influence follows from the low modulus of elasticity of the glass fibre sheet when compared to that of carbon fibre [22,39].
- Comparable ductility was obtained for the series with the same cross-sectional reinforcement ratio, and covering the side faces did not produce a demonstrable change in this parameter. The largest increase in ductility was 126.14%.
- The U-shaped configuration unified the failure mode: all five specimens failed by crushing of the timber in the compression zone, and no debonding of the sheet was recorded in this series. Rupture of the composite was not observed in any of the tested beams, which indicates that the tensile capacity of the sheet was never fully mobilised and explains why successive layers ceased to be effective.
- The numerical model and the transformed cross-section method predicted the stiffness coefficient with a mean absolute deviation from the experimental values of 10.5% and 9.4%, respectively. The accuracy obtained for the maximum load was lower.

Where the ultimate limit state governs the design, concentrating the reinforcement on the bottom face of the beam is the more effective solution, and it also gives the higher elastic stiffness. The U-shaped configuration does not increase the capacity at the same reinforcement ratio, but it makes the failure mode uniform and eliminates debonding of the sheet, which is relevant wherever the predictability of the response is important. The choice of layout should therefore follow from whether capacity or reliability governs the particular application.

The conclusions are based on five specimens per series, which limits the statistical power of the comparisons; the smallest difference in maximum load detectable with a power of 80% was 10.27 kN, that is, 54% of the mean capacity of the unreinforced beams. Non-significant results should accordingly be read as an absence of evidence rather than as evidence of absence. Further work should address the minimum extent of side coverage required to maintain the uniform failure mode, the strengthening of the compression zone, which governed failure in the most heavily reinforced series, and the verification of the observed trends on a larger number of specimens, the long-term behaviour of the strengthened members, in particular creep and the durability of the adhesive bond, and the non-destructive grading of the timber before the specimens are allocated to the series.

### Limitations of the Study

It should be noted that while extremely defective wood was rejected during visual sorting, a detailed mapping of local defects (e.g., individual knot size and location) was outside the scope of this study. The natural, residual variability of the timber is considered the primary reason for the observed discrepancies, such as the lower initial stiffness of the BG1 series. This limitation highlights the necessity of employing non-destructive testing (NDT) in future research to better evaluate the initial state of each beam.

## Figures and Tables

**Table 1:** Selected mechanical and physical properties of pine wood.

Parameter	MV	Std. Dev.	COV (%)
Global modulus of elasticity in bending, *E_m_*_,*g*,*mean*_ [GPa] *	9.48	0.82	8.64
Bending strength, *f_m_*_,*mean*_ [MPa] *	56.22	7.15	12.72
Volumetric density, *ρ_mean_* [kg/m^3^] *	533.98	47.04	8.81
Moisture content, *w* [%] *	9.74	0.83	8.52
Tensile strength parallel to grain, *f_t_*_,0,*mean*_ [MPa] **	33.73	-	-
Tensile strength perpendicular to grain, *f_t_*_,90,*mean*_ [MPa] **	0.60	-	-
Compressive strength parallel to grain, *f*_c,0,*mean*_ [MPa] **	30.65	-	-
Compressive strength perpendicular to grain, *f*_c,90,*mean*_ [MPa] **	3.73	-	-
Shear strength, *f_v_*_,*mean*_ [MPa] **	3.80	-	-

* Obtained experimentally. ** Estimated from the mean bending strength using the relations given in EN 384; these values are therefore estimates of the mean strengths of the batch tested and not characteristic values, and they are used in this sense in the numerical model described in Section 2.3. MV—mean value; Std. Dev.—standard deviation; COV—coefficient of variation.

**Table 2:** Selected mechanical and physical parameters of GFRP sheet [15].

Parameter	Value
Modulus of elasticity, *E_frp_* [GPa]	73
Tensile strength, *f_t_*_,*f*_ [MPa]	3400
Sheet mass in longitudinal direction/on area unit *m_f_* [g/m^2^]	800/880
Density, *ρ_f_* [kg/m^3^]	2600
Elongation at rupture, *ε_f_* [%]	4.5
Design thickness (estimated using mass in longitudinal direction), *t_f_* [mm]	0.308—one layer; 0.616—two layers; 0.924—three layers

**Table 3:** Selected mechanical and physical properties of adhesive [20,21].

Parameter	Value
Bending strength *f_m_*_,*mean*_ [MPa]	85.3 *
Modulus of elasticity *E_k_* [MPa]	3200
Density *ρ_k_* [kg/m^3^]	1200–1300 (1150 *)
Compressive strength *f_c_*_,*k*_ [MPa]	100

* Based on own experimental research [21].

**Table 4:** Elastic constants [18,25,26].

*E*_1_	*E*_2_	*E*_3_	*G*_12_	*G*_13_	*G*_23_	*ν*_12_	*ν*_13_	*ν*_23_
9630	320	320	600	600	60	0.34	0.34	0.46

Elastic and shear moduli are in MPa.

**Table 5:** *R_ij_* constants assumed for the FEA.

*R*_11_	*R*_22_	*R*_33_	*R*_12_	*R*_13_	*R*_23_
1.0	0.122	0.122	0.215	0.215	0.077

**Table 6:** Statistical data for the maximum load *F*.

Series	Individual Results, *F* (kN)	MV (kN)	Increases (%)	Std. Dev. (kN)	COV (%)	Group *
BN	21.47, 18.33, 14.80, 21.23, 19.50	19.07	-	2.71	14.2	a
BG1	25.92, 21.27, 21.77, 20.16, 19.56	21.74	+14.00	2.50	11.5	ab
BG2	25.07, 27.12, 28.85, 35.27, 29.15	29.09	+52.54	3.82	13.1	bc
BG3	33.17, 23.75, 39.60, 30.22, 24.11	30.17	+58.20	6.63	22.0	c
BGU1	19.94, 24.72, 25.73, 30.27, 22.18	24.57	+28.84	3.90	15.9	abc

Designations: MV—mean value; Std. Dev.—standard deviation; COV—coefficient of variation. * Homogeneous groups obtained from the ANOVA test. Statistical estimations were performed on non-rounded values.

**Table 7:** Statistical data for the deflection *u* at maximum load.

Series	Individual Results, *u* (mm)	MV (mm)	Change (%)	Std. Dev. (mm)	COV (%)	Group *
BN	35.93, 32.24, 26.04, 39.88, 24.56	31.73	-	6.48	20.4	c
BG1	37.87, 38.22, 38.81, 29.76, 51.43	39.22	+23.60	7.77	19.8	bc
BG2	37.79, 38.32, 71.41, 70.03, 62.18	55.95	+76.32	16.71	29.9	ab
BG3	71.33, 65.86, 67.86, 65.74, 39.04	61.97	+95.30	13.01	21.0	a
BGU1	38.91, 33.31, 55.76, 35.86, 35.80	39.93	+25.84	9.07	22.7	bc

Designations: MV—mean value; Std. Dev.—standard deviation; COV—coefficient of variation. * Homogeneous groups obtained from the ANOVA test. Statistical estimations were performed on non-rounded values.

**Table 8:** Statistical data for the bending stiffness coefficient *k*.

Series	Individual Results, *k* (kN/mm)	MV (kN/mm)	Increase (%)	Std. Dev. (kN/mm)	COV (%)	Group *
BN	0.72, 0.72, 0.63, 0.63, 0.84	0.71	-	0.085	12.1	a
BG1	0.87, 0.75, 0.66, 0.67, 0.48	0.68	−3.14	0.140	20.5	a
BG2	0.84, 0.87, 0.75, 0.98, 0.79	0.85	+19.64	0.087	10.3	a
BG3	0.91, 0.72, 1.08, 0.86, 0.67	0.85	+19.87	0.161	19.0	a
BGU1	0.61, 0.90, 0.72, 0.85, 0.74	0.76	+8.21	0.114	15.0	a

Designations: MV—mean value; Std. Dev.—standard deviation; COV—coefficient of variation. * Homogeneous groups obtained from the ANOVA test. Statistical estimations were performed on non-rounded values.

**Table 9:** Loads corresponding to the serviceability deflection limits *L*/300 and *L*/250.

Series	Load at *L*/300 (kN)	MV (kN)	Std. Dev. (kN)	Increase (%)	Load at *L*/250 (kN)	MV (kN)	Std. Dev. (kN)	Increase (%)
BN	3.49, 3.46, 3.00, 2.97, 3.98	3.38	0.416	-	4.16, 4.14, 3.64, 3.58, 4.79	4.06	0.489	-
BG1	4.18, 3.68, 3.27, 3.20, 2.37	3.34	0.668	−1.18	4.97, 4.31, 3.98, 3.84, 2.84	3.99	0.776	−1.82
BG2	4.04, 4.22, 3.79, 4.64, 3.75	4.09	0.363	+20.95	4.82, 5.01, 4.45, 5.54, 4.49	4.86	0.445	+19.69
BG3	4.34, 3.46, 5.03, 4.23, 3.16	4.04	0.744	+19.64	5.23, 4.14, 6.10, 5.02, 3.84	4.87	0.903	+19.79
BGU1	2.96, 4.32, 3.35, 3.88, 3.48	3.60	0.520	+6.45	3.52, 5.18, 4.07, 4.75, 4.19	4.34	0.641	+6.89

**Table 10:** Statistical data for the ductility index *µ_E_*.

Series	Individual Results, *µ_E_* (-)	MV (-)	Increase (%)	Std. Dev. (-)	COV (%)	Group *
BN	1.27, 2.36, 1.30, 1.38, 1.53	1.57	-	0.45	29.0	b
BG1	2.03, 3.14, 1.89, 1.00, 2.58	2.13	+35.86	0.80	37.7	ab
BG2	1.88, 2.75, 2.53, 2.62, 3.53	2.66	+69.90	0.59	22.1	ab
BG3	4.28, 4.61, 3.40, 3.61, 1.81	3.54	+126.14	1.09	30.6	a
BGU1	2.89, 4.14, 2.71, 1.80, 2.93	2.89	+84.63	0.83	28.9	ab

Designations: MV—mean value; Std. Dev.—standard deviation; COV—coefficient of variation. * Homogeneous groups obtained from the ANOVA test. Statistical estimations were performed on non-rounded values.

**Table 11:** Failure mode recorded for the individual specimens.

Series	Failure Mode	Timber Crushing	Composite Debonding
BN	T, T, T, T, T	0/5	NA
BG1	C+D, C+D, T+D, S+D, T+D	2/5	5/5
BG2	T+D, C, T+D, S, C	2/5	2/5
BG3	C+T+D, C+T+D, C, C, C	5/5	2/5
BGU1	C, C, C, C, C	5/5	0/5

Designations: T—cracking of the timber in the tension zone; S—shear failure of the timber; C—crushing of the timber in the compression zone; D—debonding of the composite sheet; NA—not applicable. Modes combined with a plus sign occurred in the same specimen; the modes are listed in the order in which they were observed.

**Table 12:** Comparison of the experimental and predicted values of the bending stiffness coefficient *k*.

Series	Experimental Mean (kN/mm)	Transformed Section (kN/mm)	FEM (kN/mm)
BN	0.71	0.62 (−12.7%)	0.63 (−11.3%)
BG1	0.68	0.67 (−1.5%)	0.66 (−2.9%)
BG2	0.85	0.73 (−14.1%)	0.71 (−16.5%)
BG3	0.85	0.77 (−9.4%)	0.75 (−11.8%)
BGU1	0.76	0.69 (−9.2%)	0.68 (−10.5%)

Designation: FEM—finite element method. Relative error was estimated based on the rounded values.

**Table 13:** Comparison of experimental and numerical maximum load *F*.

Series	Experimental Mean (kN)	FEM Maximum Load (kN)	FEM Mod (kN)
BN	19.07	15.31 (−19.7%)	18.95 (−0.6%)
BG1	21.74	17.74 (−18.4%)	21.92 (0.8%)
BG2	29.09	18.98 (−34.8%)	24.19 (−16.8%)
BG3	30.17	19.87 (−34.1%)	25.34 (−16.0%)
BGU1	24.57	18.24 (−25.8%)	22.43 (−8.7%)

Designation: FEM—finite element method. Relative error was estimated based on the rounded values.

## Data Availability

The original contributions presented in this study are included in the article. Further inquiries can be directed to the corresponding author.
